# Supplementary material for: CD4 derived double negative T cells prevent the development and progression of nonalcoholic steatohepatitis
Source: Nat Commun. 2021 Jan 28;12:650. doi: 10.1038/s41467-021-20941-x (PMC7844244; doi:10.1038/s41467-021-20941-x)
Supplement: Supplementary file 1 — Supplementary Information [file 41467_2021_20941_MOESM1_ESM.pdf]

Supplementary Information

Supplementary Figures

Figure S1

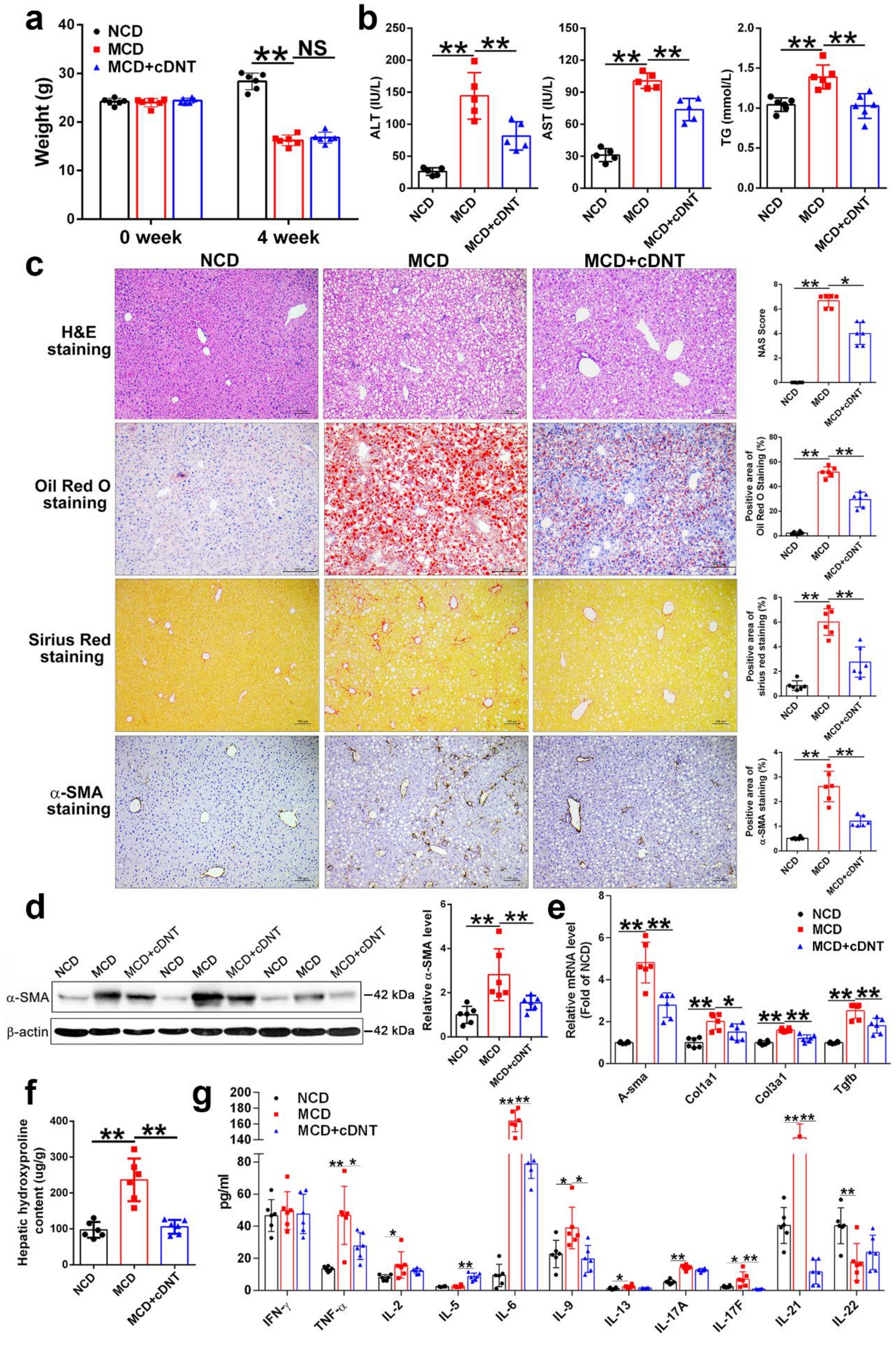

Figure S1. cDNT transfer prevented NASH development in MCD-fed mice.

C57BL/6 mice received single CD45.1-positive cDNT adoptive transfer were fed with an MCD for 4 weeks. **a** Body weight of mice from each group with or without cDNT transfer. NCD, black; MCD, red; MCD + cDNT, blue. Actual p values (left→right): 6.07e-9, 0.77. **b** Plasma ALT, AST, and TG levels were measured in NCD- and MCD-fed mice. Actual p values (left→right): 0.000018, 0.0045, 4.26e-8, 0.00056, 0.0065, 0.0058. **c** Representative H&E staining, oil red o staining, Sirius red staining and α-SMA staining in liver paraffin sections. Quantification of liver histology staining. Scale bars, 100 μm. (n = 6 biologically independent samples per group). Actual p values (left→right): for NAS score, 0.0017, 0.010; for oil red o staining, 5.81e-9, 5.69e-7; for sirius red staining, 4.03e-7, 0.000092; for α-SMA staining, 2.62e-7, 0.000034. **d** Western blot analysis of α-SMA in liver tissues of MCD-fed mice. Actual p values (left→right): 4.16e-7, 0.000061. **e** Fibrosis-related genes in the mouse liver were detected via quantitative real-time PCR. Actual p values (left→right): 1.37e-7, 0.00022, 0.00036, 0.044, 0.000001, 0.00014, 7.57e-7, 0.0024. **f** Hydroxyproline levels in liver tissues from each group. Actual p values (left→right): 0.000038, 0.000078. **g** Inflammatory cytokine levels were measured in plasma from NCD- and MCD-fed mice. Actual p values (left→right): 0.00042, 0.029, 0.037, 0.000004, 5.80e-9, 6.84e-9, 0.036, 0.013, 0.019, 1.61e-8, 0.024, 0.0035, 0.000015, 7.26e-7, 0.0084. Data are presented as the mean ± SD; n = 6 mice/group. Statistical analysis for the first right figure in **c** (NAS score group) was performed by Kruskal-Wallis multiple comparisons test, and others were performed by one-way ANOVA with post-hoc multiple comparisons test. Two-sided p values < 0.05 were considered significant. *p < 0.05; **p < 0.01; NS, not significant. Source data, including exact p values, are provided as a Source data file.

Figure S2

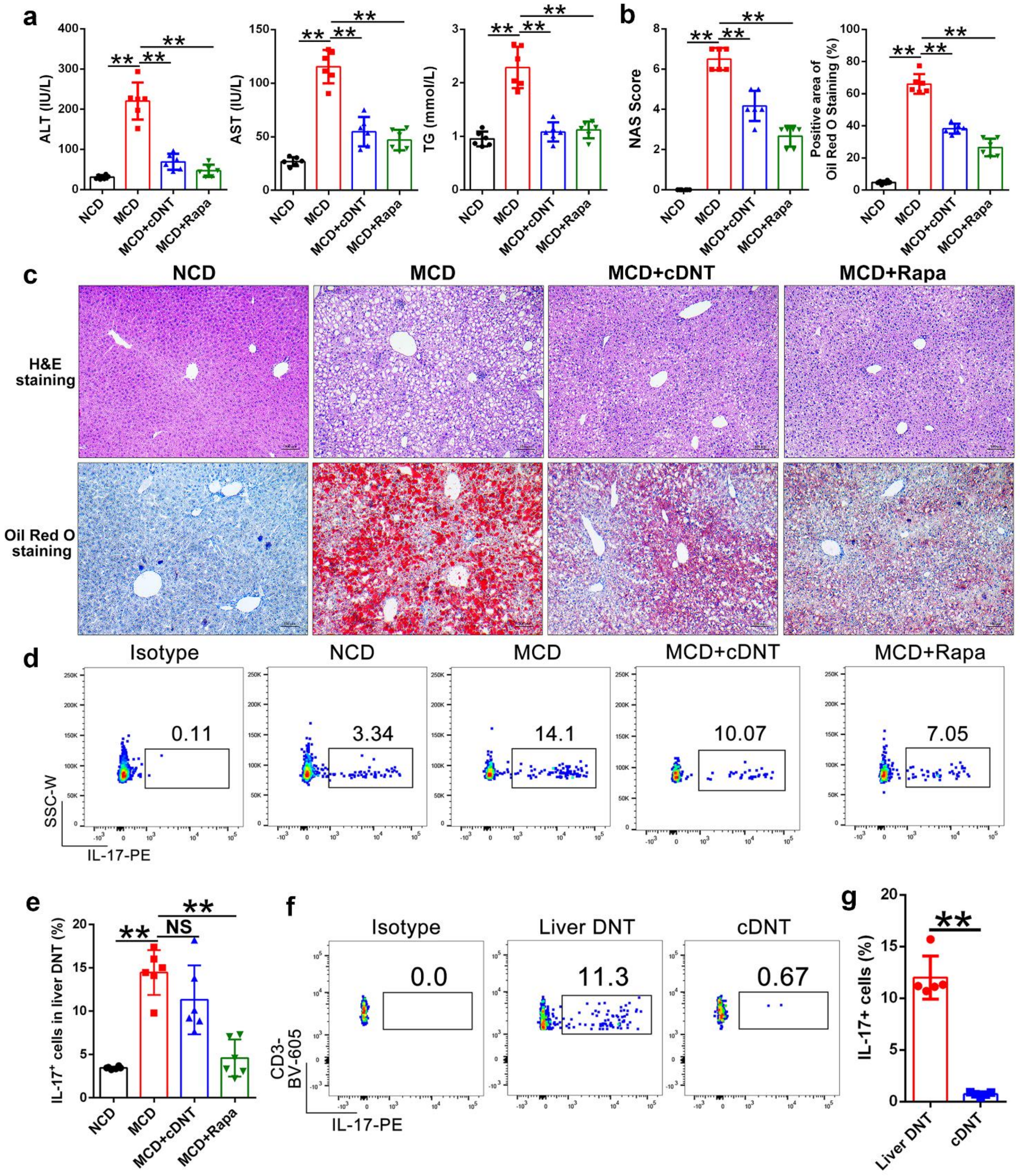

Figure S2. cDNT and Rapamycin had similar therapeutic effects on NASH.

a Plasma ALT, AST, and TG levels were measured (n = 6 mice/group). Actual p values (left→right): 4.05e-10, 2.05e-8, 1.90e-9, 1.32e-10, 8.82e-8, 1.2e-8, 2.76e-8, 1.47e-7,

2.42e-7. **b** Statistical analysis of NAS score and positive area of oil red o staining in each group (n = 6 mice/group). Actual p values (left→right): 0.0053, 0.0095, 0.0086, 8.46e-13, 3.79e-9, 7.66e-12. **c** Representative H&E staining and oil red o staining in liver paraffin sections. Scale bars, 100 μm. (n = 6 biologically independent samples per group). **d** and **e** Representative flow cytometry plots (**d**) and statistical analysis (**e**) of the percentages of IL-17^{+} cells among liver resident DNT after cDNT or rapamycin treatment (n = 6 mice/group). Actual p values (left→right): **e** 0.000002, 0.000012. **f** and **g** Representative flow cytometry plots (**f**) and statistical analysis (**g**) of the percentages of IL-17^{+} cells among liver resident DNT and cDNT in MCD-fed mice (n = 5 mice/group). For **g**: **p = 0.000002. Data are presented as the mean ± SD. Statistical analysis for the first right figure in **b** (NAS score group) was performed by Kruskal-Wallis multiple comparisons test, statistical analysis for **g** was performed by Student's t test, and others were performed by one-way ANOVA with post-hoc multiple comparisons test. Two-sided p values < 0.05 were considered significant. **p < 0.01; NS, not significant. Source data, including exact p values, are provided as a Source data file.

Figure S3

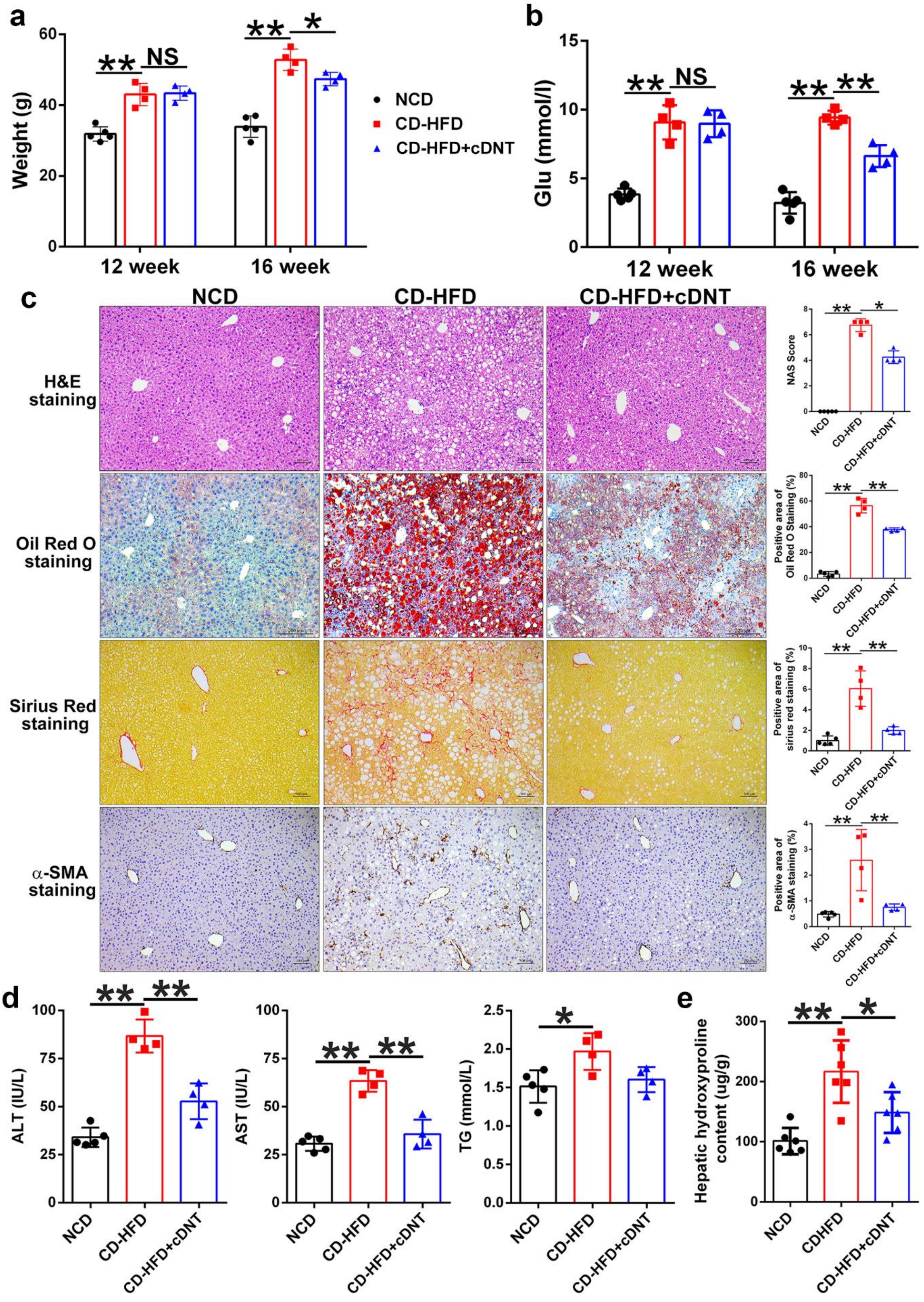
a
Weight (g)
60
40
20
0
12 week
16 week
**
NS
**
*
NCD
CD-HFD
CD-HFD+cDNT
b
Glu (mmol/l)
15
10
5
0
12 week
16 week
**
NS
**
**
c
NCD
CD-HFD
CD-HFD+cDNT
H&E staining
Oil Red O staining
Sirius Red staining
α-SMA staining
NAS Score
Positive area of Oil Red O Staining (%)
Positive area of sirius red staining (%)
Positive area of α-SMA staining (%)
d
ALT (IU/L)
AST (IU/L)
TG (mmol/L)
100
75
50
25
0
2.5
2.0
1.5
1.0
0.5
0.0
e
Hepatic hydroxyproline content (ug/g)
300
200
100
0
NCD
CD-HFD
CD-HFD+cDNT
CDHFD

Figure S3. cDNT transfer prevented NASH development in CD-HFD-fed mice. C57BL/6 mice were fed a CD-HFD for 12 weeks, received a transfer of cDNT, and were then continuously fed the CD-HFD for another 4 weeks. **a** Body weight of mice from each group before (12 weeks) and after (16 weeks) cDNT transfer. NCD, black; CD-HFD, red; CD-HFD + cDNT, blue. Actual p values (left→right): 0.00012, 0.97, 0.000003, 0.043. **b** Fasting plasma glucose levels were examined in mice fed a CD-HFD for 12 or 16 weeks. Actual p values (left→right): 0.000017, 0.99, 4.08e-7, 0.00066. **c** Representative and statistical analysis of H&E staining, oil red o staining, Sirius red staining and α-SMA staining in liver paraffin sections. Quantification of liver histology staining. Scale bars, 100 μm. Actual p values (left→right): for NAS score, 0.00617, 0.046; for oil red o staining, 1.62e-8, 0.000038; for Sirius red staining, 0.000055, 0.00050; for α-SMA staining, 0.0020, 0.0073. **d** Plasma ALT, AST, and TG levels were measured in NCD- and CD-HFD-fed mice. Actual p values (left→right): 0.000003, 0.00024, 0.000016, 0.00011, 0.020. **e** Hydroxyproline levels in liver tissues of each group. Actual p values (left→right): 0.00026, 0.018. Data are presented as the mean ± SD. NCD group, n = 5 mice/group; CD-HFD group, n = 4 mice/group; CD-HFD + cDNT group, n = 4 mice/group. Statistical analysis for the first right figure in **c** (NAS score group) was performed by Kruskal-Wallis multiple comparisons test, and others were performed by one-way ANOVA with post-hoc multiple comparisons test. Two-sided p values < 0.05 were considered significant. *p < 0.05; **p < 0.01; NS, not significant. Source data, including exact p values, are provided as a Source data file.

Figure S4

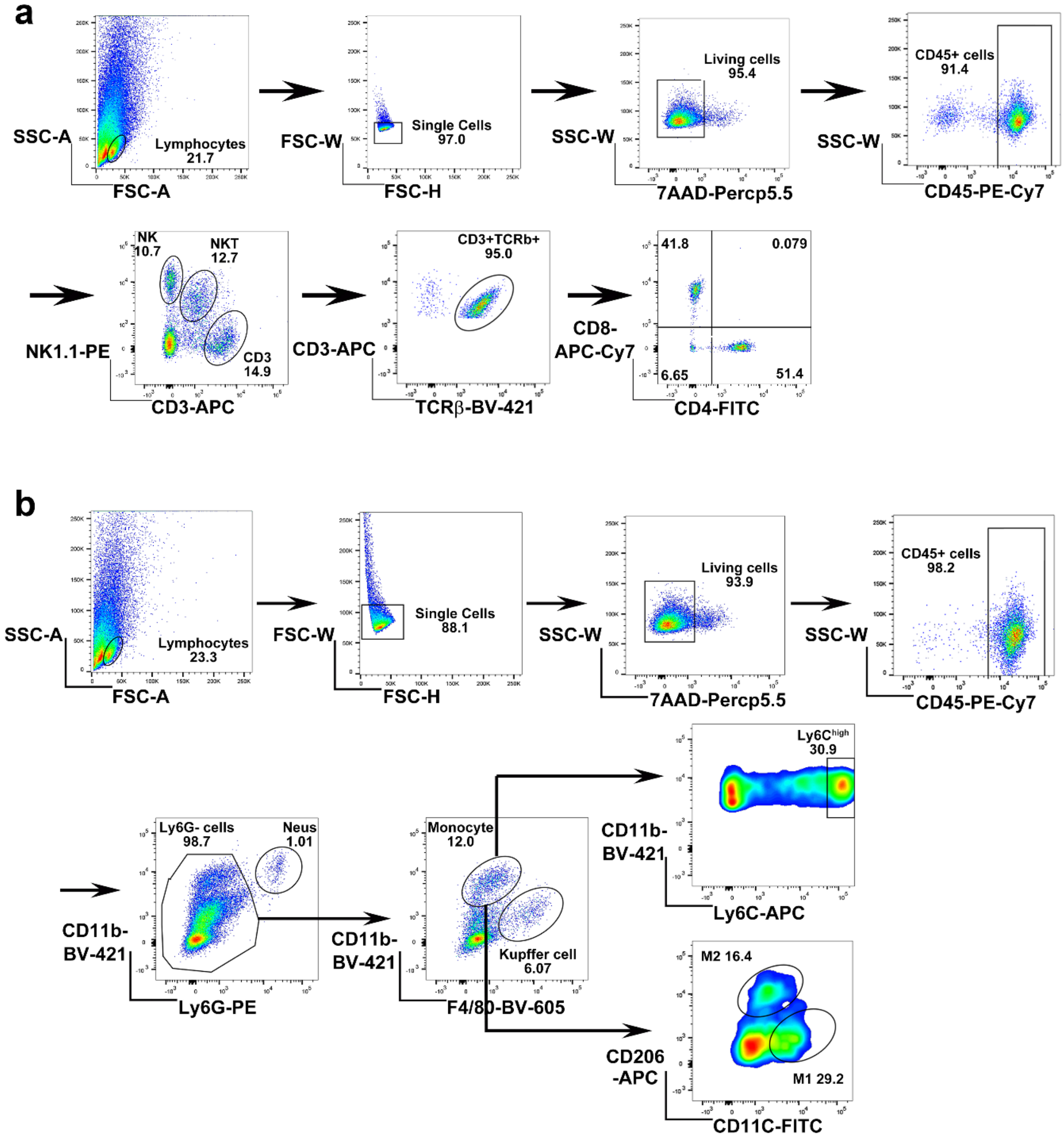

Figure S4. Representative flow cytometry images of the gating strategy used for flow cytometry analysis.

a Typical flow cytometry gating strategy to distinguish hepatic lymphocytes. **b** Typical flow cytometry gating strategy to distinguish hepatic macrophages and Kupffer cells.

Figure S5

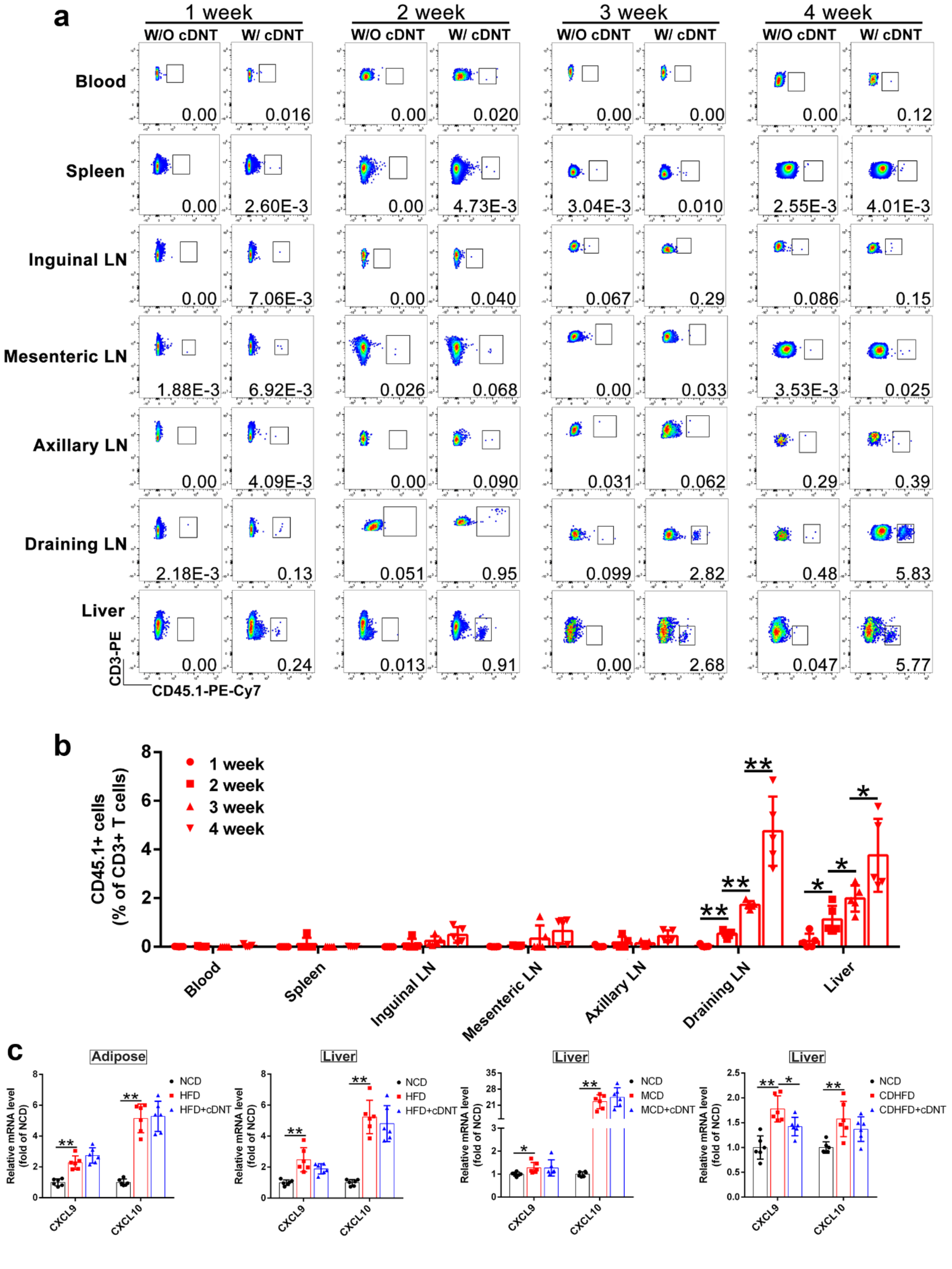

Figure S5. The tissue distribution of transferred CD45.1^{+} cDNT in MCD-fed mice.

a and **b** Representative flow cytometry plots (**a**) and statistical analysis (**b**) of the percentages of CD45.1$^+$ cDNT in blood, spleen, liver and different lymph nodes of MCD-fed mice every week. Actual p values (left→right): **b** 0.0057, 0.000008, 0.000032, 0.015, 0.029, 0.019. **c** Relative mRNA levels of *CXCL9* and *CXCL10* in liver and adipose tissues of NCD-, HFD-, MCD-, and CD-HFD-fed mice. Actual p values (left→right): 0.00018, 4.78e-7, 0.00034, 0.000003, 0.037, 1.21e-8, 0.000088, 0.043, 0.0042. Data are presented as the mean ± SD; n ≥ 5 mice/group. Statistical analysis was performed by one-way ANOVA with post-hoc multiple comparisons test. Two-sided p values < 0.05 were considered significant. *p < 0.05; **p < 0.01. Source data, including exact p values, are provided as a Source data file.

Figure S6

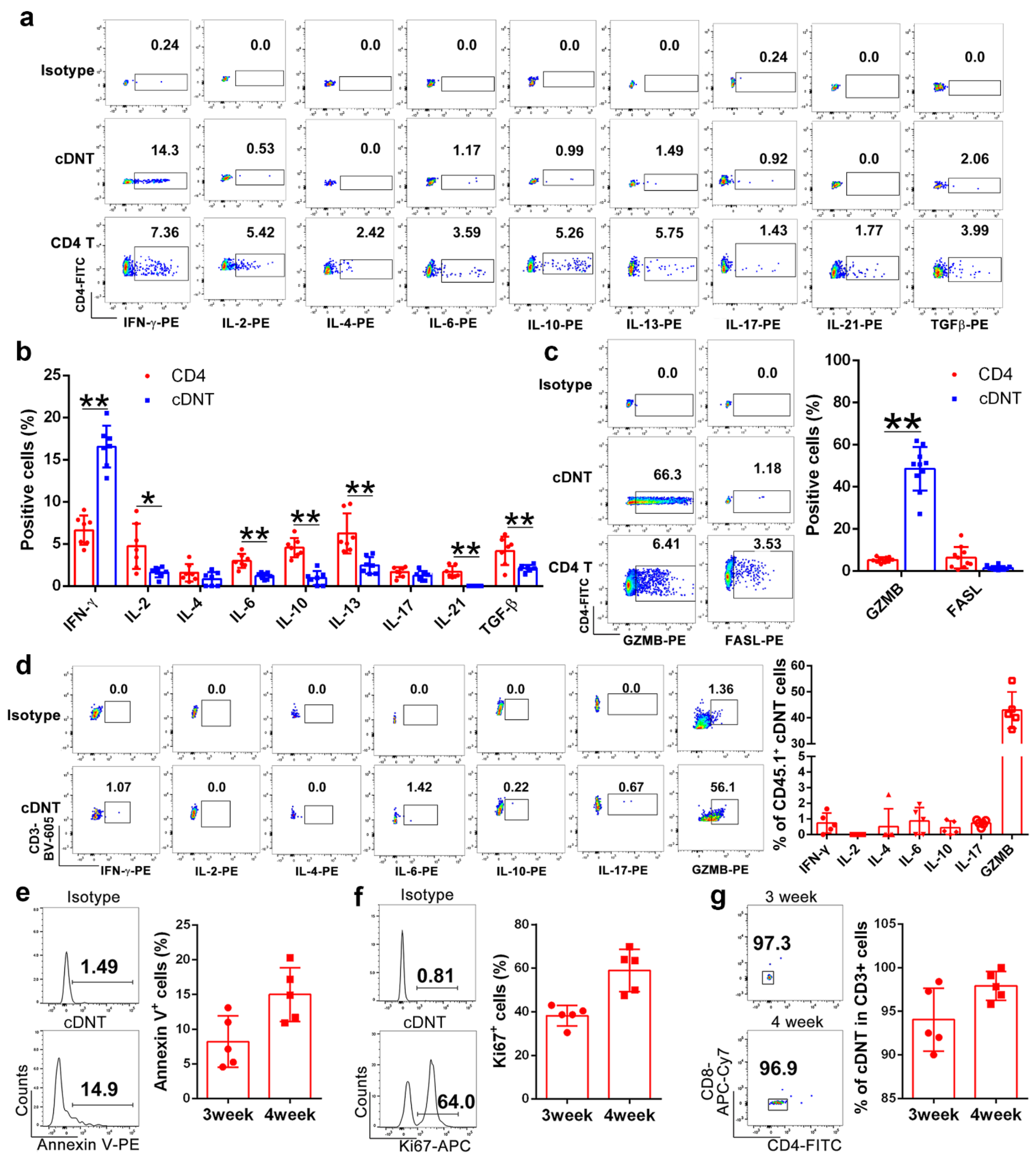

Figure S6. The cytokines secretion and survival of cDNT *in vitro* and *in vivo*.

a-c The cytokine secretion and immunoregulatory molecules expression profile and statistical analysis of activated $CD4^+$ T cells (red) and cDNT (blue) *in vitro*. (n ≥ 7

biologically independent samples per group). Actual p values (left→right): **b** 0.000002, 0.012, 0.00023, 0.000021, 0.0022, 0.00060, 0.0084; **c** 1.11e-10. **d** Representative flow cytometry plots and statistical analysis of the cytokine secretion and Granzyme B (GZMB) expression in transferred cDNT *in vivo*. (n = 5 biologically independent samples per group). **e** and **f** The Annexin V and Ki67 staining of transferred cDNT in liver tissues (n = 5 biologically independent samples per group). **g** CD4 and CD8 staining of cDNT after transfer at 3 and 4 weeks (n = 5 biologically independent samples per group). Data are presented as the mean ± SD. Statistical analysis was performed by one-way ANOVA with post-hoc multiple comparisons test. Two-sided p values < 0.05 were considered significant. *p < 0.05; **p < 0.01. Source data, including exact p values, are provided as a Source data file.

Figure S7

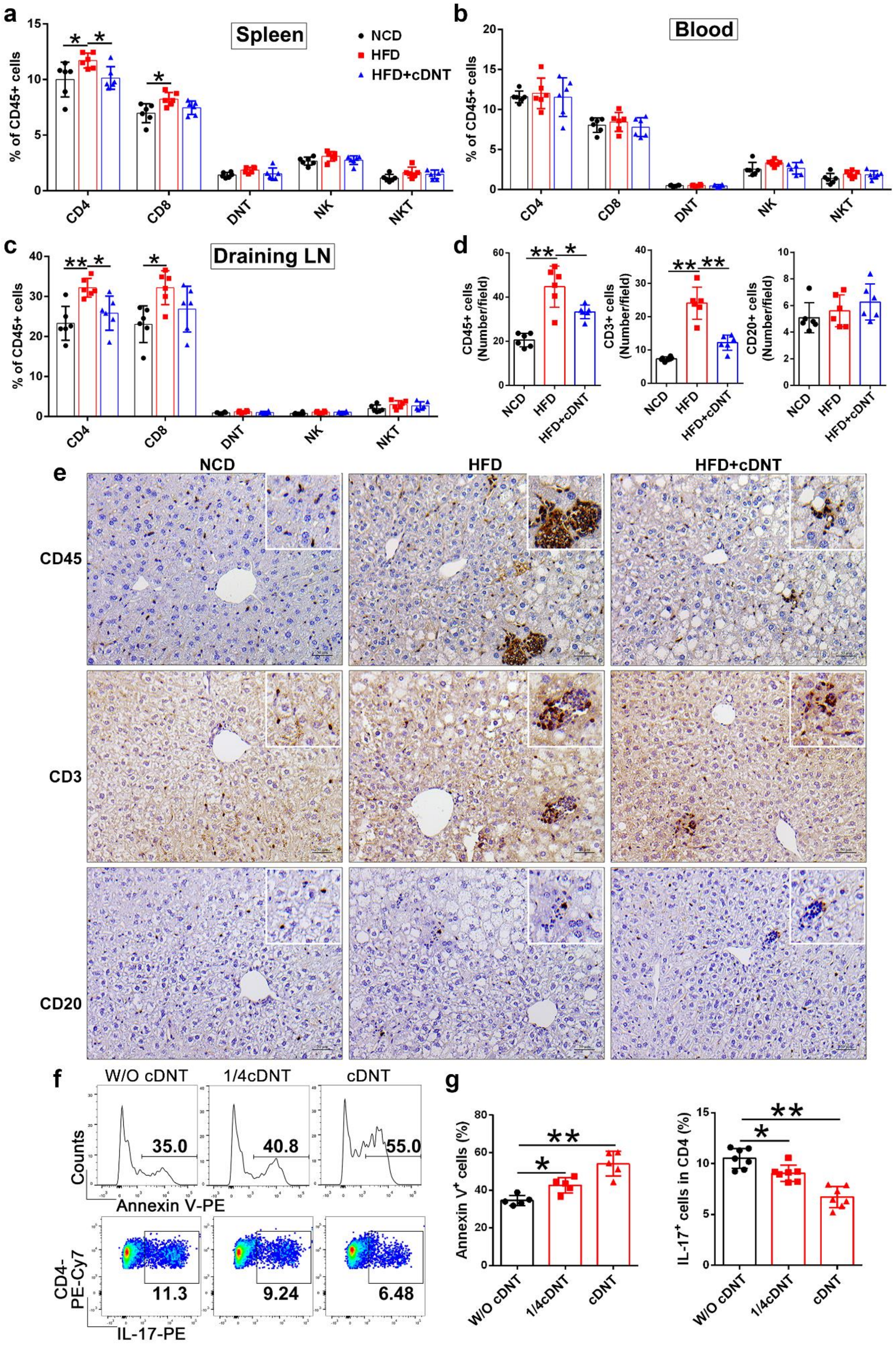

a
Spleen
NCD
HFD
HFD+cDNT
% of CD45+ cells
CD4
CD8
DNT
NK
NKT
b
Blood
c
Draining LN
d
CD45+ cells (Number/field)
CD3+ cells (Number/field)
CD20+ cells (Number/field)
e
CD45
CD3
CD20
f
W/O cDNT
1/4cDNT
cDNT
Counts
Annexin V-PE
35.0
40.8
55.0
CD4-PE-Cy7
IL-17-PE
11.3
9.24
6.48
g
Annexin V+ cells (%)
IL-17+ cells in CD4 (%)

Figure S7. The proportions of lymphocytes in different tissues of HFD-fed mice and the suppression of Th17 cells by cDNT *in vitro*.

a-c Percentages of $CD4^+$ T-cells, $CD8^+$ T-cells, DNT, NK cells, and NKT cells relative to the total number of $CD45.2^+$ cells in spleen, blood and draining lymph nodes (n = 6 mice/group). NCD, black; HFD, red; HFD + cDNT, blue. Actual p values (left→right): **a** 0.024, 0.022, 0.017; **c** 0.0023, 0.025, 0.014. **d** and **e** Representative images (**e**) and statistical analysis (**d**) of CD45, CD3, CD20 staining in liver paraffin sections of HFD-fed mice in each group (n = 6 mice/group). Scale bars, 100 μm. Actual p values (left→right): **d** 0.000011, 0.012, 3.43e-7, 0.000022. **f** and **g** Naïve $CD45.1^+$ $CD4^+$ T cells were induced to Th17 for 3 days and cocultured with $CD45.2^+$ cDNT for 24 hours. Representative flow cytometry plots (**f**) and statistical analysis (**g**) of Annexin V^+ cells and IL-17^+ cells relative to the total $CD45.1^+CD4^+$ T cells in each group (n = 5 biologically independent samples per group). Actual p values (left→right): **g** 0.046, 0.0064, 0.024, 0.00055. Data are presented as the mean ± SD. Statistical analysis was performed by one-way ANOVA with post-hoc multiple comparisons test. Two-sided p values < 0.05 were considered significant. *p < 0.05; **p < 0.01. Source data, including exact p values, are provided as a Source data file.

Figure S8

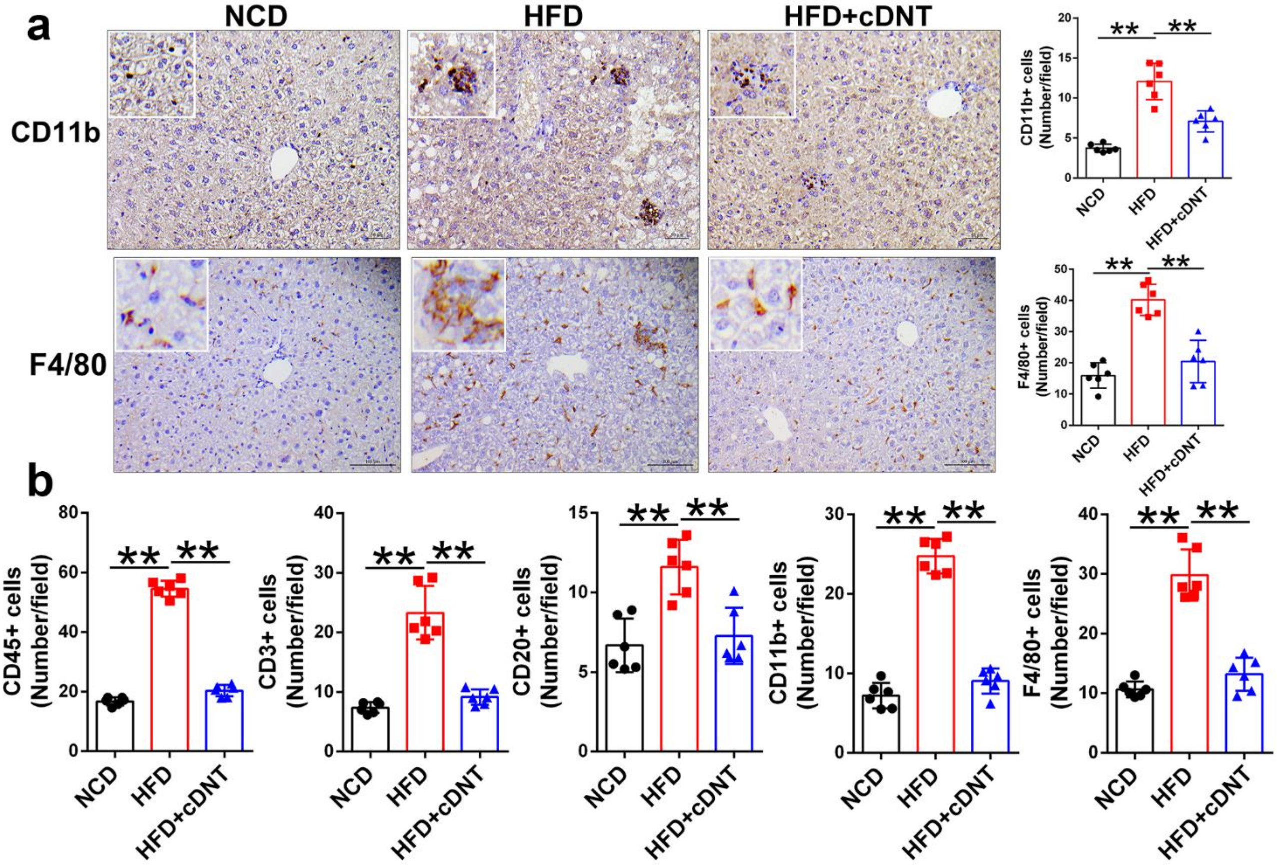

Figure S8. The immunohistochemistry of different immune cells in liver and VAT of NASH models.

a Representative images and statistical analysis of CD11b and F4/80 staining in liver paraffin sections of HFD-fed mice in each group. Scale bars, 100 μm. Actual p values (left→right): 3.62e-7, 0.00015, 0.000004, 0.000038. **b** Quantification of CD45, CD3, CD20, CD11b and F4/80 staining in VAT paraffin sections of each group. Actual p values (left→right): 5.80e-9, 5.80e-9, 1.40e-7, 6.30e-7, 0.00048, 0.0015, 5.89e-9, 6.25e-9, 5.29e-8, 3.17e-7. Data are presented as the mean ± SD; n = 6 mice/group. Statistical analysis was performed by one-way ANOVA with post-hoc multiple comparisons test. Two-sided p values < 0.05 were considered significant. *p < 0.05; **p < 0.01. Source data, including exact p values, are provided as a Source data file.

Figure S9

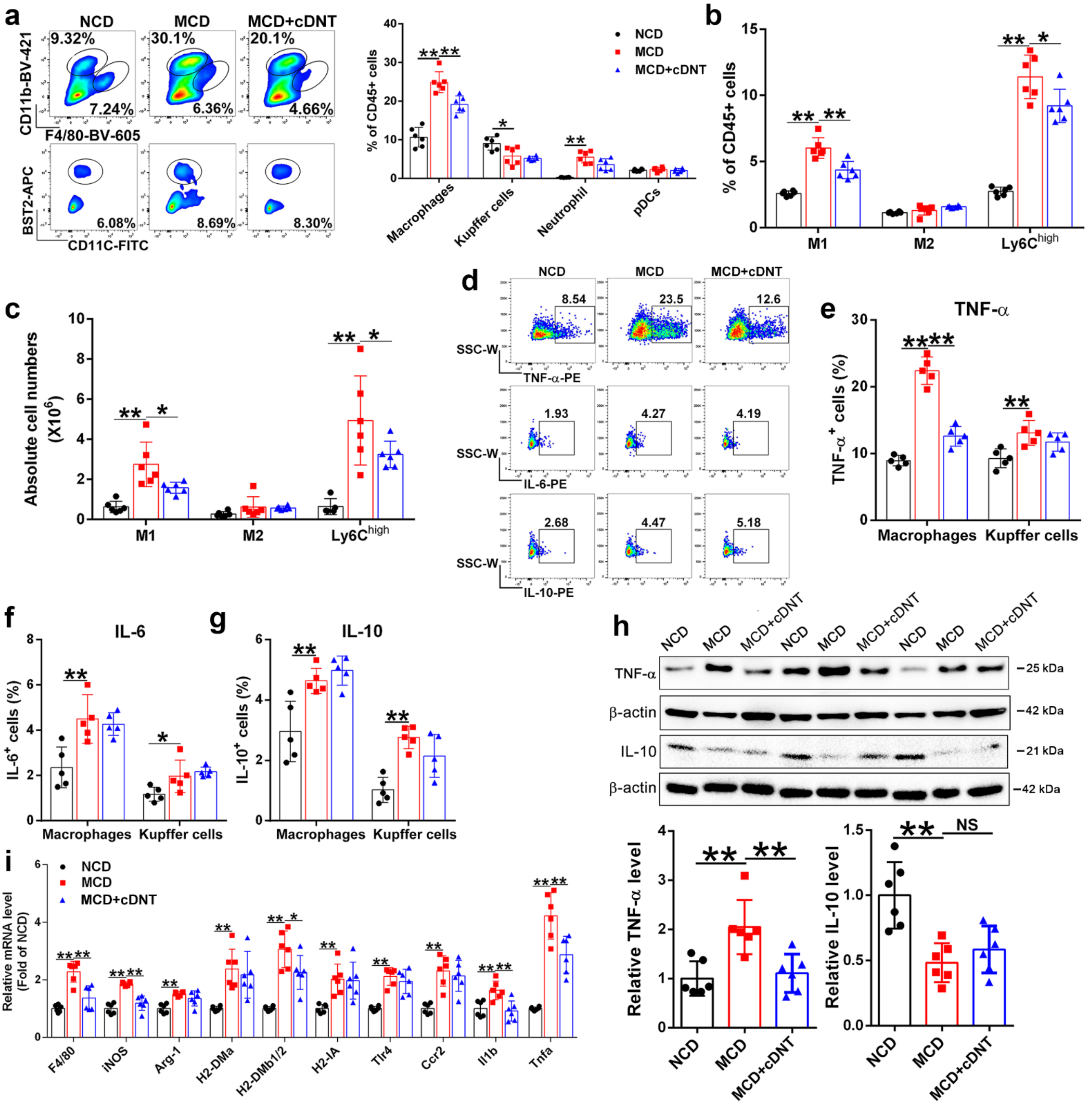

Figure S9. cDNT decreased the proportion of infiltrating macrophages and the secretion of proinflammatory cytokines in MCD-fed mice.

a Representative flow cytometry plots (left) and statistical analysis (right) of macrophages, Kupffer cells, neutrophils, and pDCs relative to intrahepatic CD45.2^{+}

cells in each group. NCD, black; MCD, red; MCD + cDNT, blue. Actual p values (left→right): 1.55e-7, 0.0030, 0.012, 0.000003. **b** Statistical analysis of M1 and M2 macrophages and $Ly6C^{high}$ cells (%$CD45.2^{+}$ cells) in mouse livers from each group, as determined by flow cytometry. Actual p values (left→right): 1.63e-7, 0.00060, 1.41e-8, 0.018. **c** Absolute numbers of M1 and M2 macrophages and $Ly6C^{high}$ cells in mouse livers from each group. Actual p values (left→right): 0.00019, 0.023, 0.00012, 0.046. **d-g** Representative flow cytometry plots (**d**) and statistical analysis of the percentages of TNF-α^{+} (**e**), IL-6^{+} (**f**), and IL-10^{+} (**g**) cells among macrophages and Kupffer cells from livers of NCD- and MCD-fed mice with or without cDNT. Actual p values (left→right): **e** 3.18e-8, 8.22e-7, 0.0059; **f** 0.0051, 0.049; **g** 0.0059, 0.00052. **h** Western blot analysis of TNF-α, IL-10 in liver tissues of MCD-fed mice. Actual p values (left→right): 0.0024, 0.0056, 0.0012, 0.66. **i** Relative mRNA levels of the indicated genes in livers from NCD- or MCD-fed mice. Actual p values (left→right): 0.000019, 0.000659, 0.000002, 0.000032, 0.00060, 0.0045, 0.000010, 0.036, 0.0068, 0.000034, 0.00038, 0.0092, 0.0037, 9.24e-8, 0.00049. Data are presented as the mean ± SD; $n \geq 5$ mice/group. Statistical analysis was performed by one-way ANOVA with post-hoc multiple comparisons test. Two-sided p values < 0.05 were considered significant. *p < 0.05; **p < 0.01; NS, not significant. Source data, including exact p values, are provided as a Source data file.

Figure S10

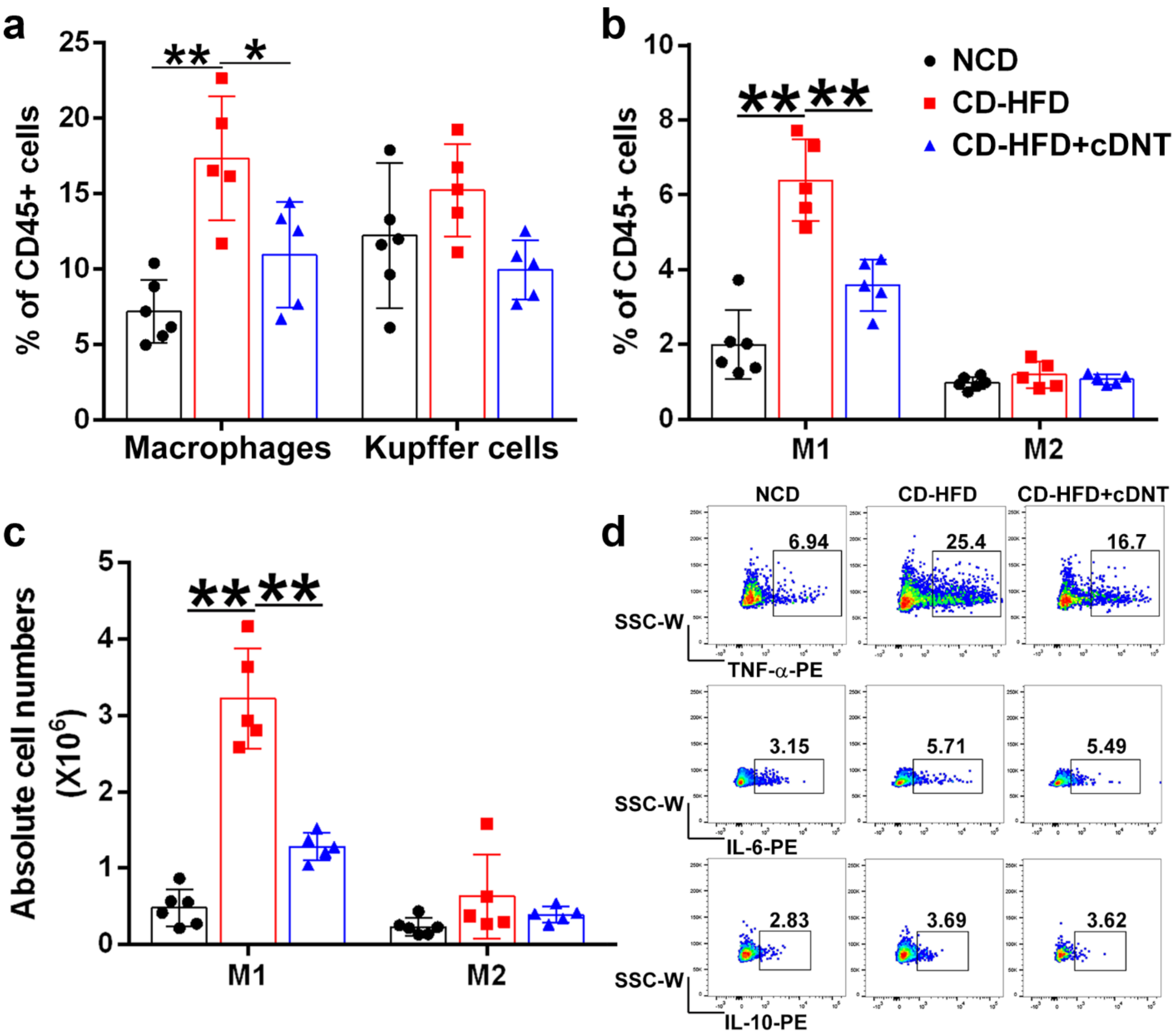

Figure S10. cDNT transfer decreased the proportion of infiltrating macrophages and the secretion of proinflammatory cytokines in CD-HFD-fed mice.

a Statistical analysis of the percentages of intrahepatic macrophages and Kupffer cells among CD45.2$^+$ cells in each group. NCD, black; CD-HFD, red; CD-HFD + cDNT, blue. Actual p values (left→right): 0.000516, 0.021. **b** Statistical analysis of M1 and M2 macrophages (%CD45.2$^+$ cells) in the livers of mice from each group, as determined by flow cytometry. Actual p values (left→right): 0.000007, 0.00087. **c** Absolute numbers of M1 and M2 macrophages in mouse livers from each group. Actual

p values (left→right): 1.42e-7, 0.000011. **d** The secretion of TNF-α, IL-6, and IL-10 by infiltrating macrophages was determined by flow cytometry. Data are presented as the mean ± SD; n = 5 mice/group. Statistical analysis was performed by one-way ANOVA with post-hoc multiple comparisons test. Two-sided p values < 0.05 were considered significant. *$p < 0.05$; **$p < 0.01$. Source data, including exact p values, are provided as a Source data file.

Figure S11

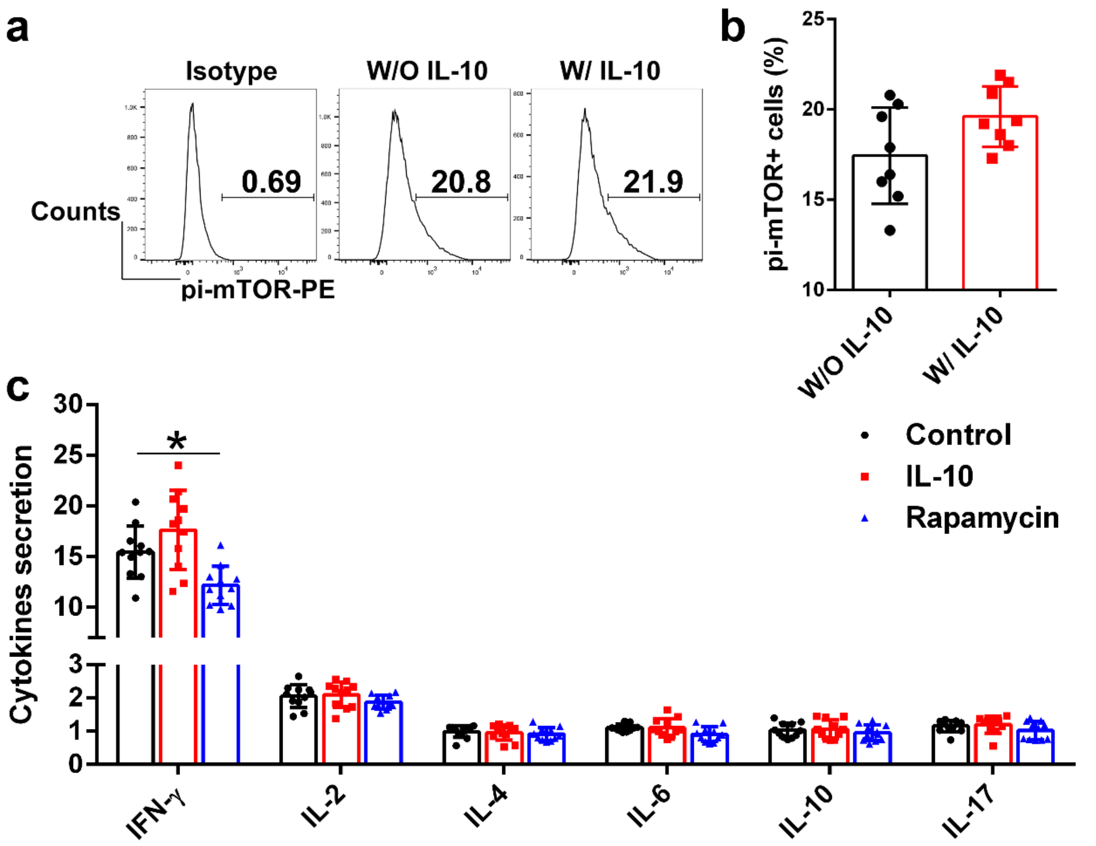

Figure S11. The expression of mTOR pathway after IL-10 stimulation and the roles of mTOR pathway in cDNT cytokine secretion.

a and **b** Representative flow cytometry plots (**a**) and statistical analysis (**b**) of pi-mTOR expression in cDNT with or without IL-10 stimulation (n = 8 biologically independent samples per group). **c** The cytokines secretion from cDNT cells after IL-10 (red) or rapamycin (blue) stimulation (n = 11 biologically independent samples per group). *p = 0.032. Statistical analysis was performed by one-way ANOVA with post-hoc multiple comparisons test. Two-sided p values < 0.05 were considered significant. Source data, including exact p values, are provided as a Source data file.

Supplementary Table S1. Primer sequences used for real-time PCR

Gene	Strand	Primer sequence (5'-3')
a-SMA	Sense	*ATCGTCCACCGCAAATGC*
	Antisense	*AAGGAACTGGAGGCGCTG*
Col1a1	Sense	*CTGCTGGCAAAGATGGAGA*
	Antisense	*ACCAGGAAGACCCTGGAATC*
Col3a1	Sense	*CAAATGGCATCCCAGGAG*
	Antisense	*CATCTCGGCCAGGTTCTC*
TGFb	Sense	*GAGGTCACCCGCGTGCTA*
	Antisense	*TGTGTGAGATGTCTTTGGTTTTCTC*
Tbx21	Sense	*GTTCCCATTCCTGTCCTTCA*
	Antisense	*ACCCACTTGCCGCTCTG*
Gata3	Sense	*GGGTTCGGATGTAAGTCGAG*
	Antisense	*CCACAGTGGGGTAGAGGTTG*
RORγT	Sense	*TACCTTGGCCAAAACAGAGG*
	Antisense	*ATGCCTGGTTTCCTCAAAA*
Foxp3	Sense	*CACCCAGGAAAGACAGCAACC*
	Antisense	*GCAAGAGCTCTTGTCCATTGA*
IL-4	Sense	*AGC AGT TCC ACA GGC ACA AG*
	Antisense	*AGC AGT TCC ACA GGC ACA AG*
IL-15	Sense	*CATCCATCTCGTGCTACTTGTG*
	Antisense	*GCCTCTGTTTTAGGGAGACCT*
IL-17	Sense	*GCTCCAGAAGGCCCTCAGACT*
	Antisense	*CCAGCTTTCCCTCCGCATTGA*
Ifng	Sense	*GGCCATCAGCAACAACATAAGCGT*
	Antisense	*TGGGTTGTTGACCTCAAACTTGGC*
F4/80	Sense	*CTTTGGCTATGGGCTTCCAGTC*
	Antisense	*GCAAGGAGGACAGAGTTTATCGTG*
Inos	Sense	*ATCTTTGCCACCAAGATGGCCTGG*
	Antisense	*TTCCTGTGCTGTGCTACAGTTCCG*
Arg-1	Sense	*TGACTGAAGTAGACAAGCTGGGGAT*
	Antisense	*CGACATCAAAGCTCAGGTGAATCGG*
H2-Dma	Sense	*TGAAGGTCAAATCCCAGTGTCC*
	Antisense	*AGCGGTCAATCTCGTGTGTCAC*
H2-DMb1/2	Sense	*CAACAAGGAGAAGACGGCTCA*
	Antisense	*CGCTGTGCTGAACCACG*
H2-IA-a	Sense	*AGGCTTCCTGAGTTTGG*
	Antisense	*AGGGTGTTGGGCTGAC*
TLR4	Sense	*ACCTGGCTGGTTTACACGTC*
	Antisense	*CTGCCAGAGACATTGCAGAA*
CCR2	Sense	*TTTGTTTTTGCAGATGATTCAA*
	Antisense	*TGCCATCATAAAGGAGCCAT*
Tnfa	Sense	*TCCCAGGTTCTCTTCAAGGGA*

	Antisense	*GGTGAGGAGCACGTAGTCGG*
IL-10	Sense	*AGAAAAGAGAGCTCCATCATGC*
	Antisense	*TTATTGTCTTCCCGGCTGTACT*
IL4Ra	Sense	*AGCGGGCACGATAACT*
	Antisense	*CTACCACCTGACCACCAA*
IL6Ra	Sense	*CCCTTGTCAACGCCATCT*
	Antisense	*AAACAGCACAGCCTTCG*
IL10Ra	Sense	*GCCTCACGACTTCTTCC*
	Antisense	*CCTCCAGGTCCACGAT*
IL17Ra	Sense	*CAGCATCACCGTAAGCG*
	Antisense	*CCTCACAGTCAGGCACAA*
Ifngr1	Sense	*TTGACGAGCACTGAGGA*
	Antisense	*AGGAACCCGAATACACC*
TNFR1	Sense	*GACCGGGAGAAGAGGGATAG*
	Antisense	*GTTCCTTTGTGGCACTTGGT*
TNFR2	Sense	*GTCCAGAATCTCCCTCCTT*
	Antisense	*CAGCCTGCCTGTAACCT*
Bcl-2	Sense	*GGAAGGTAGTGTGTGTGG*
	Antisense	*ACTCCACTCTCTGGGTTCTTGG*
Bcl-xl	Sense	*AACATCCCAGCTTCACATAACCCC*
	Antisense	*GCGACCCCAGTTTACTCCATCC*
Cytc	Sense	*CACGCTTTACCCTTCGTTCT*
	Antisense	*CTCATTTCCCTGCCATTCTCTA*
EpCAM	Sense	*AGGGGCGATCCAGAACAACG*
	Antisense	*ATGGTCGTAGGGGCTTTCTC*
Cflar	Sense	*TGGAATACCGTGACAGTC*
	Antisense	*CTTGCATATCGGCGAAC*
Spi1	Sense	*TCAGATGAGGAGGAGGGTG*
	Antisense	*TTGGACGAGAACTGGAAGG*
NKG2A	Sense	*ACTCATTGCTGGTACCCTGGG*
	Antisense	*GAGGACAAGGCTGTGCTGAAG*
NKG2D	Sense	*ACGTTTCAGCCAGTATTGTGC*
	Antisense	*GGAAGCTTGGCTCTGGTTC*
Prf1	Sense	*CTGCCACTCGGTCAGAATG*
	Antisense	*CGGAGGGTAGTCACATCCAT*
NKp46	Sense	*CACAGGAGGTGTTGAGAA*
	Antisense	*AAGAAGTAGGGTCGGTAG*
KLRG1	Sense	*TCTCATCCCTTCCTCTGC*
	Antisense	*TTGCGTCTTTCTGTCTTGT*
Slamf6	Sense	*CAGCTAATGAATGGCGTTCTAGG*
	Antisense	*CTTAGGTTGATAACGAGGGCAG*
Sh2d1b2	Sense	*ATGGTGGTTCATCTTTCA*
	Antisense	*TTCAGTCTTCTCGCTCC*
CD94	Sense	*GGCAGTTTCTAGGATCACTCG*
	Antisense	*CTTCCTGGAATTCTACAGTGGT*

CD107a	Sense	*AGGCTCCACTGATTTGACT*
	Antisense	*TGCTCCCGTTTGCTTC*
CD226	Sense	*ACCACATGGCTTTCTTGCTC*
	Antisense	*CAGCATGAGAGTTGGACCAG*
Fasl	Sense	*TGAATTACCCATGTCCCCAG*
	Antisense	*AAACTGACCCTGGAGGAGCC*
Lair1	Sense	*GCTGGTTTGCCTTATC*
	Antisense	*AGTGGTCCTCTGGGTAT*
GAPDH	Sense	*AAGGTCATCCCAGAGCTGAA*
	Antisense	*CTGCTTCACCACCTTCTTGA*
CXCL9	Sense	*TGTGGAGTTCGAGGAACCCT*
	Antisense	*TGCCTCGGCTGGTGCTG*
CXCL10	Sense	*ATCCCTGCGAGCCTATC*
	Antisense	*GCCATCCACTGGGTAAA*

Supplementary Materials and Methods

Reagents and antibodies

The LEGENDplex Mouse Th Cytokine Panel was purchased from BioLegend (CA, USA). Mouse T-cell enrichment columns were obtained from R&D Systems (MN, USA). Anti-PE microbeads and magnetic bead separation columns were obtained from Miltenyi Biotec (Bergisch Gladbach, Germany). ALT, AST, and TG detection kits were purchased from NanJing JianCheng Biochemical Institute (Jiang Su, China). Collagenase IV and bovine serum albumin (BSA) were obtained from Sigma (MO, USA). Percoll was purchased from GE Healthcare (USA). Fluorochrome-conjugated antibodies against mouse CD3, CD4, CD8, CD11b, CD11C, CD25, CD45.1, CD45.2, CD69, CD206, Ter119, B220, Ly6C, Ly6G, MHC II, NK1.1, TCRαβ, Foxp3, IL-6, IL-10, IL-17, IFN-γ, TNF-α, PD-1, Tim3, and CTLA4 were obtained from BioLegend. Antibodies against B-cell lymphoma 2 (Bcl-2) and B-cell lymphoma-extra large (Bcl-xL) were purchased from Cell Signaling Technology (MA, USA). Recombinant mouse IL-2, IL-4, IL-6, IL-10, IL-17, IFN-γ, TNF-α, and granulocyte-macrophage colony-stimulating factor (GM-CSF) were obtained from Peprotech (NJ, USA). Mouse IL-10 neutralizing antibody was obtained from R&D Systems, anti-mouse CD210 (IL-10R) neutralizing antibody was obtained from BioLegend, and mouse Qa-1b neutralizing antibody was purchased from BD Biosciences (CA, USA). The Annexin V-PE kit was purchased from BD Biosciences, the EdU staining kit was purchased from RiboBio Corporation (Guangzhou, China), and the Caspase-3 staining kit was purchased from Beyotime Biotechnology (Shanghai, China).
