# Supplementary material for: CD4 derived double negative T cells prevent the development and progression of nonalcoholic steatohepatitis
Source: Nat Commun. 2021 Jan 28;12:650. doi: 10.1038/s41467-021-20941-x (PMC7844244; doi:10.1038/s41467-021-20941-x)
Supplement: Supplementary file 3 — Reporting Summary [file 41467_2021_20941_MOESM3_ESM.pdf]

nature research

Corresponding author(s): Dong Zhang

Last updated by author(s): Nov 11, 2020

Reporting Summary

Nature Research wishes to improve the reproducibility of the work that we publish. This form provides structure for consistency and transparency in reporting. For further information on Nature Research policies, see Authors & Referees and the Editorial Policy Checklist .

Statistics

For all statistical analyses, confirm that the following items are present in the figure legend, table legend, main text, or Methods section.

n/a	Confirmed	
☐	☒	The exact sample size (*n*) for each experimental group/condition, given as a discrete number and unit of measurement
☐	☒	A statement on whether measurements were taken from distinct samples or whether the same sample was measured repeatedly
☐	☒	The statistical test(s) used AND whether they are one- or two-sided<br>*Only common tests should be described solely by name; describe more complex techniques in the Methods section.*
☒	☐	A description of all covariates tested
☐	☒	A description of any assumptions or corrections, such as tests of normality and adjustment for multiple comparisons
☐	☒	A full description of the statistical parameters including central tendency (e.g. means) or other basic estimates (e.g. regression coefficient) AND variation (e.g. standard deviation) or associated estimates of uncertainty (e.g. confidence intervals)
☐	☒	For null hypothesis testing, the test statistic (e.g. *F*, *t*, *r*) with confidence intervals, effect sizes, degrees of freedom and *P* value noted<br>*Give P values as exact values whenever suitable.*
☒	☐	For Bayesian analysis, information on the choice of priors and Markov chain Monte Carlo settings
☒	☐	For hierarchical and complex designs, identification of the appropriate level for tests and full reporting of outcomes
☒	☐	Estimates of effect sizes (e.g. Cohen's *d*, Pearson's *r*), indicating how they were calculated

Our web collection on statistics for biologists contains articles on many of the points above.

Software and code

Policy information about availability of computer code

Data collection	BD FACSDiva (6.1.3) was used to collect flow cytometric data.
Data analysis	FlowJo (v10.0.2) was used for flow cytometric analysis. Graphpad Prism (v6.02) and ImageJ software (1.42q) were used for statistical analysis.

For manuscripts utilizing custom algorithms or software that are central to the research but not yet described in published literature, software must be made available to editors/reviewers. We strongly encourage code deposition in a community repository (e.g. GitHub). See the Nature Research guidelines for submitting code & software for further information.

Data

Policy information about availability of data

All manuscripts must include a data availability statement. This statement should provide the following information, where applicable:

- Accession codes, unique identifiers, or web links for publicly available datasets
- A list of figures that have associated raw data
- A description of any restrictions on data availability

All data generated and supporting the findings of this study are available within the paper. Gene Expression Omnibus (accession no. GSE134346) is deposited at GEO database, and is released publicly.

Field-specific reporting

Please select the one below that is the best fit for your research. If you are not sure, read the appropriate sections before making your selection.

Life sciences study design

All studies must disclose on these points even when the disclosure is negative.

Sample size	A reasonable sample size was chosen based on our previous studies and similar literatures to ensure adequate reproducibility of results.
Data exclusions	None.
Replication	Experimental findings were successfully reproduced in at least three independent experiments.
Randomization	Animals were randomly allocated into experimental groups.
Blinding	We were blinded to group allocation during data collection and/or analysis.

Reporting for specific materials, systems and methods

We require information from authors about some types of materials, experimental systems and methods used in many studies. Here, indicate whether each material, system or method listed is relevant to your study. If you are not sure if a list item applies to your research, read the appropriate section before selecting a response.

Materials & experimental systems

n/a	Involved in the study	
☐	☒	Antibodies
☒	☐	Eukaryotic cell lines
☒	☐	Palaeontology
☐	☒	Animals and other organisms
☒	☐	Human research participants
☒	☐	Clinical data

Methods

n/a	Involved in the study	
☒	☐	ChIP-seq
☐	☒	Flow cytometry
☒	☐	MRI-based neuroimaging

Antibodies

Antibodies used

The following antibodies were used for flow cytometry:
B-cell lymphoma 2(Bcl-2) BioLegend #633508 clone BCL/10C4 1:100
B220 BioLegend #103207 clone RA3-6B2 1:100
CD3 BioLegend #100306 #100312 clone 145-2C11 1:200
CD4 BioLegen #100406 #100422 #100434 #100428 clone GK1.5 1:200
CD8 BioLegend # 100734 #100712 # 100705 clone 53-6.7 1:200
CD11b BioLegend #101236 clone M1/70 1:200
CD11C BioLegend #117306 clone N418 1:200
CD25 BioLegend # 101904 clone 3C7 1:200
CD45.1 BioLegend #110730 clone A20 1:200
CD45.2 BioLegend #109814 clone 104 1:200
CD69 BioLegend #104514 #104507 clone H1.2F3 1:200
CD206 BioLegend #141708 clone C068C2 1:200
Ter119 BioLegend #116208 clone TER-119 1:200
GzmB BioLegend #515403 clone GB11 1:100
Ly6C BioLegend #128016 clone HK1.4 1:200
Ly6G BioLegend #127654 clone 1A8-Ly6g 1:200
MHC II BioLegend #107614 clone M5/114.15.2 1:200
NK1.1 BioLegend #108708 clone PK136 1:200
TCRb BioLegend #109208 clone H57-597 1:200
Foxp3 BioLegend #126404 clone FJK-165 1:100
IL-6 BioLegend #504504 clone MP5-20F3 1:100
IL-10 BioLegend #50500 clone JES5-16E3 1:100
IL-17 BioLegend #50690 clone eBio17B7 1:100
IFN-γ BioLegend #505808 clone XMG1.2 1:100
TNF-a BioLegend #506306 clone MP6-XT22 1:100
NKG2A BioLegend #142803 clone 16A11 1:100
NKG2D BioLegend #115605 clone A10 1:100
B-cell lymphoma-extra large (Bcl-xL) Cell Signaling Technology((MA, USA) #13835 clone 54H6 1:100

The following antibodies were used for Neutralization:
Mouse IL-10 neutralizing antibody R&D Systems #MAB417-100 clone JES052A5 0.1ug/ml, 1.0ug/ml
Mouse CD210 (IL-10R) neutralizing antibody BioLegend #112706 clone 1B1.3a 1ug/ml, 10ug/ml
Mouse Qa-1b neutralizing antibody BD Biosciences (CA, USA) #566640 clone 6A8.6F10.1A6 10ug/ml

Validation

Bcl-2: https://www.biolegend.com/en-gb/products/pe-anti-bcl-2-antibody-6466
B220: https://www.biolegend.com/en-gb/products/pe-anti-mouse-human-cd45r-b220-antibody-447
CD3: https://www.biolegend.com/en-gb/products/fitc-anti-mouse-cd3epsilon-antibody-23
https://www.biolegend.com/en-gb/products/apc-anti-mouse-cd3epsilon-antibody-21
CD4: https://www.biolegend.com/en-gb/products/fitc-anti-mouse-cd4-antibody-248
https://www.biolegend.com/en-gb/products/pe-cyanine7-anti-mouse-cd4-antibody-1919
https://www.biolegend.com/en-gb/products/percp-cyanine5-5-anti-mouse-cd4-antibody-4220
https://www.biolegend.com/en-gb/products/pacific-blue-anti-mouse-cd4-antibody-3316
CD8: https://www.biolegend.com/en-gb/products/percp-cyanine5-5-anti-mouse-cd8a-antibody-4255
https://www.biolegend.com/en-gb/products/apc-anti-mouse-cd8a-antibody-150
https://www.biolegend.com/en-gb/products/fitc-anti-mouse-cd8a-antibody-153
CD11b: https://www.biolegend.com/en-gb/products/brilliant-violet-421-anti-mouse-human-cd11b-antibody-7163
CD11C: https://www.biolegend.com/en-gb/products/fitc-anti-mouse-cd11c-antibody-1815
CD25: https://www.biolegend.com/en-gb/products/pe-anti-mouse-cd25-antibody-129
CD45.1: https://www.biolegend.com/en-gb/products/pe-cyanine7-anti-mouse-cd45-1-antibody-4917
CD45.2: https://www.biolegend.com/en-gb/products/apc-anti-mouse-cd45-2-antibody-2759
CD69: https://www.biolegend.com/en-gb/products/apc-anti-mouse-cd69-antibody-3169
https://www.biolegend.com/en-gb/products/pe-anti-mouse-cd69-antibody-265
CD206: https://www.biolegend.com/en-gb/products/apc-anti-mouse-cd206-mmr-antibody-7425
Ter119: https://www.biolegend.com/en-gb/products/apc-anti-mouse-cd206-mmr-antibody-7425
GzmB: https://www.biolegend.com/en-gb/products/fitc-anti-human-mouse-granzyme-b-antibody-6066
Ly6C: https://www.biolegend.com/en-us/products/apc-anti-mouse-ly-6c-antibody-6047
Ly6G:https://www.biolegend.com/en-us/products/percp-anti-mouse-ly-6g-antibody-13351
MHC II: https://www.biolegend.com/en-us/products/apc-anti-mouse-i-a-i-e-antibody-2488
NK1.1: https://www.biolegend.com/en-us/products/pe-anti-mouse-nk-1-1-antibody-431
TCRb: https://www.biolegend.com/en-us/products/pe-anti-mouse-tcr-beta-chain-antibody-272
Foxp3: https://www.biolegend.com/en-us/products/pe-anti-mouse-foxp3-antibody-4660
IL-6: https://www.biolegend.com/en-us/products/pe-anti-mouse-il-6-antibody-972
IL-10: https://www.biolegend.com/en-us/products/pe-anti-mouse-il-10-antibody-944
IL-17: https://www.biolegend.com/en-us/products/pe-anti-mouse-il-17a-antibody-1633
IFN-γ: https://www.biolegend.com/en-us/products/pe-anti-mouse-ifn-gamma-antibody-997
TNF-a: https://www.biolegend.com/en-us/products/pe-anti-mouse-tnf-alpha-antibody-978
NKG2A: https://www.biolegend.com/en-us/products/pe-anti-mouse-cd159a-nkg2ab6-antibody-7543
NKG2D: https://www.biolegend.com/en-us/products/pe-anti-mouse-cd314-nkg2d-antibody-1480
Bcl-xL: https://www.cellsignal.cn/products/antibody-conjugates/bcl-xl-54h6-rabbit-mab-pe-conjugate/13835?N=4294956287&Ntt=54h6&fromPage=plp
Mouse IL-10 neutralizing antibody: https://www.rndsystems.com/cn/products/mouse-il-10-antibody-jes052a5_mab417
Mouse CD210 (IL-10R) neutralizing antibody: https://www.biolegend.com/en-us/products/pe-anti-mouse-cd210-il-10-r-antibody-1513
Mouse Qa-1b neutralizing antibody: https://www.bdbiosciences.com/cn/reagents/research/antibodies-buffers/immunology-reagents/anti-mouse-antibodies/cell-surface-antigens/pe-mouse-anti-mouse-qa-1b-6a86f101a6/p/566640

Animals and other organisms

Policy information about studies involving animals; ARRIVE guidelines recommended for reporting animal research

Laboratory animals

Eight-week-old weight-matched male C57BL/6 mice and CD45.1 congenic C57BL/6 mice were purchased from The Jackson Laboratory (ME, USA). The mice were maintained in a pathogen-free conditions, with controlled temperature (20-24oC), humidity (45-65%) and light cycle (12-h light/dark).

Wild animals

No wild animals were used in this study.

Field-collected samples

No field collected samples were used in the study.

Ethics oversight

All procedures were performed in accordance with the guidelines set by the Institutional Animal Care and Ethics Committee at Beijing Friendship Hospital.

Note that full information on the approval of the study protocol must also be provided in the manuscript.

Flow Cytometry

Plots

Confirm that:

☒ The axis labels state the marker and fluorochrome used (e.g. CD4-FITC).

☒ The axis scales are clearly visible. Include numbers along axes only for bottom left plot of group (a 'group' is an analysis of identical markers).

☒ All plots are contour plots with outliers or pseudocolor plots.

☒ A numerical value for number of cells or percentage (with statistics) is provided.

Methodology

Sample preparation	After mice were anesthetized, each mouse liver was perfused with 30 mL of normal saline (NS) through left cardiac perfusion until its liver changed to a pale color, at which point the liver was carefully removed and digested with 0.01% type IV collagenase for 30 min at 37oC. The mixture was then dissociated using a gentle-MACS dissociator (Miltenyi Biotec, Bergisch-Gladbach, Germany). The cell suspension was filtered through a 70-um nylon cell strainer and centrifuged at 50 x g for 5 min to obtain the supernatant, which was then centrifuged at 500 x g for 5 min. The cell pellet was used to detect hepatic infiltrated monocytes and Kupffer cells. The abovementioned cell pellet was resuspended in 30% Percoll in Hanks' Balanced Salt Solution (HBSS), gently overlaid onto 70% Percoll and centrifuged at 800 x g for 25 min. The cells were collected from the interface to detect intrahepatic T cells.
Instrument	FACSAriaII (BD Biosciences)
Software	FlowJo software (Tree Star)
Cell population abundance	Cell populations were sorted to >95% purity post sort in pilot experiments, as determined by flow cytometry.
Gating strategy	CD4 T cells were gated as CD3+NK1.1-CD4+CD8-, CD8 cells were gated as CD3+NK1.1-CD4-CD8+, NK cells were gated as CD3-NK1.1+, NKT cells were gated as CD3+NK1.1+. Th1 cells were gated as CD4+IFN-r+, Th2 cells were gated as CD4+IL-13+, Th17 cells were gated as CD4+IL-17+, Treg cells were gated as CD4+Foxp3+. Macrophages were gated as CD45 +CD11bhighF4/80int, Kupffer cells were gated as CD45+CD11bintF4/80high, Neutrophils cells were gated as CD45+CD11b +Ly6G+ cells, DC cells were gated as CD45+CD11C+MHC II+. M1 macrophages were gated as CD11C+CD206- cells, M2 macrophages were gated as CD11C-CD206+ cells.

☒ Tick this box to confirm that a figure exemplifying the gating strategy is provided in the Supplementary Information.
